# Oxycardiorespirograms in neonatal monitoring—current gaps and future potential of artificial intelligence: a mini-review

**DOI:** 10.3389/fped.2025.1680074

**Published:** 2025-10-07

**Authors:** Kora Helm, Alexandra Epp, Michael Gerstlauer, Mathias Kaspar, Ludwig C. Hinske, Melanie L. Conrad, Fabian B. Fahlbusch

**Affiliations:** ^1^ Neonatology and Pediatric Intensive Care, Medical Faculty, University of Augsburg, Augsburg, Germany; ^2^ Institute for Digital Medicine, University of Augsburg, Augsburg, Germany; ^3^ Pediatric Pulmonology, Medical Faculty, University of Augsburg, Augsburg, Germany; ^4^ Institute of Microbiology, Infectious Diseases and Immunology, Charité-Universitätsmedizin Berlin, Corporate Member of Freie Universität Berlin, Humboldt-Universität zu Berlin, Berlin, Germany

**Keywords:** oxycardiorespirogram, machine learning, explainability, vital signs, time series, biosignals

## Abstract

The neonatal oxycardiorespirogram (OCRG) captures synchronized, multi-channel recordings of respiratory patterns, heart rate variability, transcutaneous oxygen tension, and relative skin perfusion in neonates. As a non-invasive, point-of-care modality, OCRG is routinely used to assess cardiorespiratory stability in high-risk infants, particularly preterm neonates at risk for apnea, bradycardia, and desaturation. These events can persist beyond hospital discharge, elevating morbidity and mortality, yet no standardized tool reliably predicts which infants will experience clinically significant post-discharge episodes. Although OCRG is established in clinical practice, its rich time-series data remains largely underutilized for predictive modeling. In contrast, machine learning methods have achieved strong performance in related neonatal monitoring tasks - such as apnea detection, sepsis prediction, sleep staging, and extubation readiness - by integrating multimodal biosignals and temporal modeling strategies. These advances highlight the opportunity to apply machine learning analytics and explainability methods to OCRG data, enabling the discovery of physiological patterns, refining risk stratification, and informing individualized interventions such as the timing of caffeine withdrawal, initiation of home monitoring, or discharge planning. Given the multimodal and sequential structure of OCRGs, time-series-based machine learning, including both shallow and deep learning approaches, represents particularly promising analytic strategies for future applications. This mini-review synthesizes current gaps in OCRG-based analytics, examines transferable lessons from existing machine learning applications in neonatal biosignals, and outlines a translational roadmap for evolving OCRG from a descriptive monitoring tool into a predictive platform for precision neonatal care.

## Introduction

Cardiorespiratory instability in neonates, particularly preterm infants, remains a major challenge in neonatal intensive care units (NICUs) (1). Continuous, precise monitoring is essential not only for acute clinical decision-making, but also for discharge planning and long-term prognostication (2). The oxycardiorespirogram (OCRG) provides a non-invasive, point-of-care method to assess overall cardiorespiratory stability by capturing the dynamic interplay between respiratory effort, oxygenation, and cardiac activity in real time. By multi-channel recording of respiratory patterns, oxygen saturation, and heart rate variability, OCRG provides a more integrated physiological assessment than single-parameter monitoring, potentially enhancing the detection of subtle instabilities.

Wang et al. (3) illustrated that multi-channel OCRG can serve as a practical alternative to polysomnography for guiding respiratory support in complex neonatal cases, particularly in infants with recurrent apneic episodes during both sleep and wakefulness. Despite its clinical utility, OCRG-derived data remain conspicuously absent from current machine learning, including shallow and deep learning applications.

This underutilization contrasts sharply with parallel advances in neonatal monitoring. Numerous studies over the past decade have successfully applied machine learning to neonatal images, genetic profiles, electronic health records, and standard vital signs. Recent work has leveraged biosignals such as respiratory rate, heart rate, or their variability to predict sepsis (4), sleep stage transitions (5, 6), and weaning readiness from mechanical ventilation (7). The detection of apnea of prematurity (AOP) -affecting over 50% of preterm infants (8) - has also been enhanced by algorithmic approaches. Varisco et al. (9) demonstrated that AOP often occurs in clusters with distinctive breathing patterns that can be computationally recognized, enabling improved early-warning systems.

These advances highlight the feasibility and clinical potential of machine learning in neonatal monitoring. However, its application to OCRG remains largely unexplored. Several factors contribute to this gap, including motion artifacts, limited and heterogeneous datasets, and the absence of standardized annotation protocols. Moreover, many existing neonatal machine learning models lack explainability (XAI), a critical barrier to clinician trust and regulatory acceptance (10).

This mini-review examines the clinical relevance of OCRG for neonatal cardiorespiratory diagnostics, outlines the current evidence gap regarding machine learning integration, and discusses how combining OCRG with machine learning and XAI could transform it from a descriptive monitoring tool into a predictive and decision-supportive asset in professional neonatal care.

## Unlocking the predictive power of oxycardiorespirograms: clinical context and gaps

Cardiorespiratory instability due to central apnea is a hallmark complication in preterm neonates. Infants born before 37 weeks of gestation are at elevated risk, with nearly universal occurrence of apnea episodes among those born before 28 weeks (11). These events, often accompanied by bradycardia and oxygen desaturation (apnea–bradycardia syndrome), primarily reflect immaturity of central autonomic control. While extremely preterm infants receive caffeine citrate to stimulate respiratory drive and reduce hypoxemic episodes as the current standard-of-care, late preterm and term neonates require differential evaluation for obstructive, infectious, or neurological causes. Caffeine, currently the most commonly prescribed drug in NICUs, reduces central apnea, shortens the duration of invasive ventilation, and improves long-term neurodevelopmental outcomes (12). However, the optimal timing for caffeine discontinuation remains a subject of ongoing scientific debate, as premature withdrawal increases the risk of recurrent apnea and post-discharge cardiorespiratory events. In contrast, unnecessary prolongation can delay discharge and increase healthcare costs (11). Due to the lack of a standardized predictive test for apnea persistence, discharge readiness is often based on bedside observation and short-term monitoring.

Based on our tertiary neonatal care experience, multi-channel oxycardiorespirography is routinely applied to support decision-making regarding caffeine weaning and discharge readiness. OCRG serves as a practical complement to standardized laboratory-based polysomnography (11), which remains the gold standard for detailed sleep and respiratory assessment but is typically reserved for specialized diagnostic settings due to its complexity, resource intensity, and focus on comprehensive sleep studies rather than routine bedside monitoring. Capturing respiratory waveform and rate, instantaneous heart rate, and transcutaneous oxygen tension over four to eight hours, OCRG provides a time-resolved, multi-dimensional profile of cardiorespiratory function during both sleep and wakefulness.

In current clinical workflows, OCRG recordings are typically processed into summarized reports using proprietary software, allowing clinicians to review essential findings efficiently. While this approach supports timely decision-making, the resulting data reduction limits access to the high-resolution time-series signals, where subtle physiological dynamics may reside. This highlights the opportunity for advanced analytical approaches to augment existing OCRG workflows. By leveraging full-resolution time-series data, machine learning techniques can move beyond summary metrics to identify latent temporal and cross-channel patterns, quantify physiological variability, and derive predictive markers relevant to apnea recurrence, discharge readiness, and individualized management strategies.

## ML-driven approaches to neonatal biosignals: building blocks for OCRG innovation

Machine learning (ML) applications have advanced substantially in neonatal biosignal analysis, offering predictive insights across several physiological domains (8). Numerous studies have demonstrated the value of ML for outcomes such as apnea detection, sepsis prediction, sleep state classification, and extubation readiness, often by leveraging high-frequency or multimodal data streams from standard NICU monitoring. Kallonen et al. (4) showed that convolutional neural networks trained on high-frequency electrocardiography (ECG), respiratory impedance, and photoplethysmography could predict late-onset sepsis up to 44 h before clinical suspicion, highlighting the benefit of transforming temporal physiological signals into spectral representations. Koolen et al. (5) and Ghimatgar et al. (6) successfully classified neonatal sleep states using electroencephalography (EEG) based inputs, using support vector machines or recurrent networks, and post-processing with hidden Markov models, achieving accuracies around 80%–82%. These approaches show good performance for shallow learning and deep learning models. Additionally, they highlight that the usage of average features already achieves good accuracy. Fraiwan et al. (13) extended the sleep classification approach to multi-class sleep staging (awake, quiet, active sleep), achieving up to 95% accuracy with long short-term memory networks. Facing this high increase in accuracy compared to the other studies, they demonstrated the benefit of following a deep learning approach by using the raw signal as input, rather than aggregated features.

ML has also improved decision support for respiratory care. Shalish et al. (7) applied support vector machines to cardiorespiratory signals [ECG, chest and abdominal movement, oxygen saturation (SpO₂), plethysmography] to predict extubation success, achieving 80% identification rates. Similarly, Varisco et al. (9) utilized features from ECG, chest impedance, and SpO₂, employing logistic regression and ensemble models to predict apnea, with AUROCs ranging from 0.88 to 0.9. These studies emphasize that multimodal inputs and temporal modeling capture neonatal physiology more effectively than isolated parameters.

In most neonatal ML studies, high-frequency biosignals were not analyzed in their raw form. Instead, handcrafted feature extraction and spectral transformations were commonly employed to enhance model performance, particularly for shallow ML approaches. In contrast, deep learning methods increasingly allow more direct use of complex time-series data. These methodological strategies provide a framework for processing OCRG data while preserving time dependencies and the synchronized, multimodal nature of ORCG. To align analytic strategies more closely with these characteristics, we next outline specific time-series-based machine learning approaches that may be particularly relevant for OCRG.

### OCRG data characteristics and time-series analysis

A unique feature of OCRG recordings is their structure as synchronized, multimodal time series, combining respiratory waveforms, oxygenation, and cardiac variability across different temporal scales. This multimodal composition creates both challenges, such as heterogeneity, noise, and artifacts, and opportunities, as temporal dependencies and cross-signal coupling can be directly exploited for prediction.

Using machine learning to analyse multivariate time series data is considered one of the major challenges across domains (14, 15). For time series classification tasks, shallow learning techniques such as HIVE-COTE (16), TS-CHIEF (17), and ROCKET (18) have been state-of-the-art for many years. More recently, deep learning methods as ResNet (19) and InceptionTime (20) have begun to match or outperform these architectures, especially when trained on univariate time series data. Another notable deep learning approach is the Multivariate Time Series Transformer (21), based on the transformer architecture (22), which outperformed ROCKET on various datasets and shows high efficiency, even with fewer training samples. Recent work in multivariate time series increasingly leverages transformer-based architectures (22) or graph neural networks (23), surpassing foundational techniques such as RNNs and CNNs (15).

Beyond unimodal models, multimodal fusion strategies (early, late, or attention-based) are particularly promising for OCRG, as they can dynamically weight the contribution of different signals depending on the clinical context. To date, no published studies have applied these methods specifically to OCRG. This gap constitutes a limitation of our review but also highlights the translational potential for advancing neonatal time series analytics.

## Discussion: opportunities and translational potential

The neonatal OCRG captures a rich, multivariate profile of cardiorespiratory physiology, spanning respiratory waveforms and rate, heart rate variability, transcutaneous oxygen tension, and relative perfusion over extended monitoring periods. Although routinely used to guide clinical decisions - such as determining readiness for caffeine weaning, need for respiratory support, or hospital discharge - OCRG recordings, which typically span four to eight hours, are condensed into summarized reports for clinician review. This necessary simplification enables timely decisions but leaves much of the high-resolution temporal information underexplored. In contrast, other neonatal biosignals, including ECG, photoplethysmography, respiratory impedance, and EEG, have successfully been integrated into ML workflows to predict outcomes such as sepsis, sleep state transitions, apnea of prematurity, and extubation success (10). These advances indicate that OCRG, as a unified biosignal modality, is ideally positioned for similar ML-driven innovations.

A key opportunity lies in analyzing OCRG data beyond the summary metrics currently used in practice. The raw, high-frequency time series may contain latent temporal and cross-channel patterns, such as complex desaturation dynamics or heart rate-respiration coupling, that could refine risk prediction and thus support clinical decision making. As outlined above, OCRGs share the characteristics of complex multivariate time series, making them suitable for the architectures already used for multivariate time series classification or forecasting in other domains. Importantly, combining OCRG data with clinical variables such as gestational age, ventilation history, or neurological comorbidities has the potential to enhance model generalizability and clinical applicability. To further ensure clinical applicability and improve interpretability and trust, XAI (24) must be embedded into OCRG-based models. Techniques such as SHapley Additive exPlanations (SHAP) (25) and Integrated Gradients (26) calculate quantitative contributions of individual features (e.g., recurrent desaturation patterns or bradycardia episodes) to a model's prediction. Visualizing these values allows comparison between features deemed important by the model and those considered clinically relevant by human experts, thereby improving transparency and interpretability.

Applying ML and XAI to OCRG can therefore transform this modality from a descriptive monitoring tool into a predictive platform for neonatal care. Such integration could streamline discharge planning by predicting apnea recurrence risk, reduce unnecessary hospitalization by better timing of caffeine withdrawal, and improve individualized management of cardiorespiratory instability. The success of ML approaches in sepsis detection, sleep staging, and extubation modeling provides a methodological foundation, but OCRG's unique combination of synchronized biosignals offers distinct potential for advancing precision neonatal care.

Future progress will depend on creating standardized, annotated OCRG datasets, developing robust preprocessing pipelines, and prospectively validating predictive models across diverse neonatal populations. In addition, learning the ORCG data with different model architectures designed to solve time-series analysis, such as ROCKET, ResNet, transformer models, or graph neural networks, should be examined to identify the architecture yielding optimal performance under clinical constraints. Embedding explainability techniques will be essential to ensure transparency, regulatory acceptance, and clinician trust. By aligning OCRG-specific data characteristics with these methodological advances, future research can move beyond proof-of-concept and develop clinically actionable prediction tools.
